# Integrative Metabolomic and Transcriptomic Landscape during *Akebia trifoliata* Fruit Ripening and Cracking

**DOI:** 10.3390/ijms242316732

**Published:** 2023-11-24

**Authors:** Yongli Jiang, Yanlin Du, Chongyang Chen, Danfeng Wang, Yu Zhong, Yun Deng

**Affiliations:** 1 Faculty of Food Science and Engineering, Kunming University of Science and Technology, Kunming 650500, China; yongli_jiang0617@163.com (Y.J.); duyanlin_work@163.com (Y.D.); 19110780959@163.com (C.C.); 2 Department of Food Science and Technology, Shanghai Jiao Tong University, Shanghai 200240, Chinazhongyu@sjtu.edu.cn (Y.Z.)

**Keywords:** *Akebia trifoliata*, multi-omics, fruit cracking, cell wall, phytohormone

## Abstract

*Akebia trifoliata* fruit is prone to crack after ripening, but little is known about the mechanism underlying the cracking process. This study integrated transcriptomic and metabolomic data, revealing significant changes in 398 metabolites and 8414 genes during ripening and cracking, mainly impacting cell-wall metabolism. Multi-omics joint analysis indicated that genes related to polygalacturonase, pectate lyase, α-amylase, and glycogen phosphorylase were up-regulated after cracking, degrading cell wall and starch. Concurrently, diminished photosynthetic metabolism and heightened phenylpropanoid metabolism suggested alterations in cuticle structure, potentially impacting cell-wall robustness. Numerous auxin and abscisic acid signaling-related genes were expressed, and we assume that they contributed to the promoting peel growth. These alterations collectively might compromise peel strength and elevate expanding pressure, potentially leading to *A. trifoliata* cracking. Transcription factors, predominantly ethylene response factors and helix-loop-helix family members, appeared to regulate these metabolic shifts. These findings provide valuable insights into *A. trifoliata* cracking mechanisms; however, direct experimental validation of these assumptions is necessary to strengthen these conclusions and expedite their commercial utilization.

## 1. Introduction

*Akebia trifoliata* fruit, commonly known as “wild banana” in China, boasts a kidney-shaped, fleshy berry with green skin in its youth, maturing to a purple hue [1,2,3]. While it has been celebrated for centuries in China for its sweet and distinctive flavor, it suffers from a natural tendency to split along the ventral suture upon reaching maturity during the Chinese lunar August [4,5]. This splitting profoundly impacts their commercial viability, as it not only leads to unappealing appearances but also reduces shelf life due to oxidative stress and microbial intrusion, causing substantial financial losses in both the fresh market and processing sectors [6,7]. Thus, it is imperative to delve into the mechanisms underlying *A. trifoliata* fruit cracking, aiming to prevent it and advance industrial growth.

Fruit cracking accompanies the synthesis of a multitude of metabolites and the transcription of numerous genes [4,7]. Nowadays, several studies have focused on the transcriptional regulation of *A. trifoliata* cracking-related genes. Niu et al. [8] characterized the transcriptome of *A. trifoliata* using Illumina RNA-seq technology and conducted an integrative analysis of the transcriptome and proteome of *A. trifoliata* fruit [4]. Their research implied that cell-wall metabolism plays a crucial role in *A. trifoliata* fruit cracking. Yang et al. [9] identified a total of 106,547 unigenes, including 26 genes associated with ethylene synthesis. Fruit cracking is a complex physiological process controlled by many genes together [10]. Beyond cell-wall metabolism and ethylene synthesis, genes involved in phenylpropanoid, plant hormone, and cuticle metabolisms also play essential roles in fruit ripening and cracking [11,12]. Moreover, as the final products of the cell biological regulation process, the relative levels of metabolites can be perceived as plant responses to genetic and environmental fluctuations [13,14]. Previous studies have shown that combining untargeted metabolome analysis with high-throughput sequencing can provide insight into the ripening and cracking mechanisms of fruit, as demonstrated by Wang et al. [15] and Zhu et al. [16]. However, to date, only few studies have explored the mechanism of *A. trifoliata* fruit cracking through the integration of transcriptomic and metabolomic analyses.

Therefore, this study endeavors to unearth candidate metabolites and genes implicated in *A. trifoliata* fruit cracking by conducting a comprehensive analysis of transcriptomic and metabolomic data from fruit peels across four developmental stages (Figure S1). The observations from this study serve as a cornerstone for future investigations focused on cultivating crack-resistant varieties and preserving *A. trifoliata* fruits, which holds significant promise in advancing the *A. trifoliata* industry.

## 2. Results and Discussion

### 2.1. Alterations in Color, Chlorophyll Content, and Phytochemical Profile in the A. trifoliata Fruit Peels

**Figure 1:**
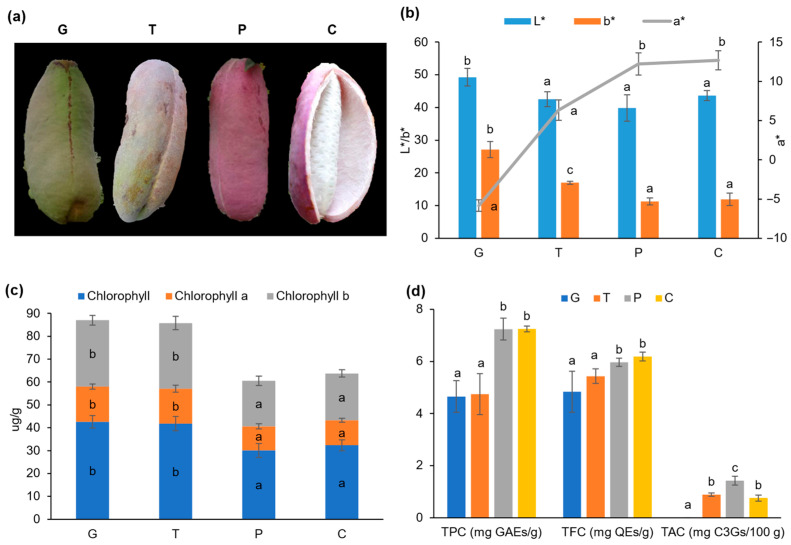
The phenotypes and physiological traits of *A. trifoliata* fruits during ripening. (**a**) The *A. trifoliata* fruit in four developmental stages. G: Green period, T: Transition period, P: Purple period, C: Cracking period. (**b**–**d**) The changes in color profile, chlorophylls content, and phytochemical profile of *A. trifoliata* fruit. Different superscripts in the same color column indicate significant differences at the *p* < 0.05 level. TPC: Total polyphenol content, TFC: Total flavonoid content, TAC: Total anthocyanin content, GAEs: gallic acid equivalents, QEs: Quercetin equivalents, C3Gs: cyanidin-3-O-glucoside equivalents. All sample analyses were conducted in triplicate.

As the fruit matured, noticeable changes in the color properties of *A. trifoliata* fruit peels were observed (Figure 1a). The L* and b* values decreased while the a* value increased, transitioning the color from green to purple (*p* < 0.05) (Figure 1b). These color shifts were discernible to the naked eye, with the overall darkening of the peel. Plant color is predominantly influenced by chlorophylls and flavonoids [17,18]. This study confirmed that the color changes were accompanied by variations in chlorophyll content, which gradually decreased in the early stages of ripening (Figure 1c). But there was no significant (*p* > 0.05) difference in the color properties of fruits between the purple and cracking periods. It can be found that TPC and TFC showed decreasing trends, but did not significantly change from the purple period to the cracking period. During ripening, TAC initially increased and significantly decreased after cracking (Figure 1d). The results showed that anthocyanins not only influenced peel color changes but were also linked to fruit cracking. However, given the complexity of this process, further research into a more precise assessment is necessary, including metabolic biomarkers, genes, and pathways.

### 2.2. Metabolome Profiling of A. trifoliata Peel over the Ripening Stages

**Figure 2:**
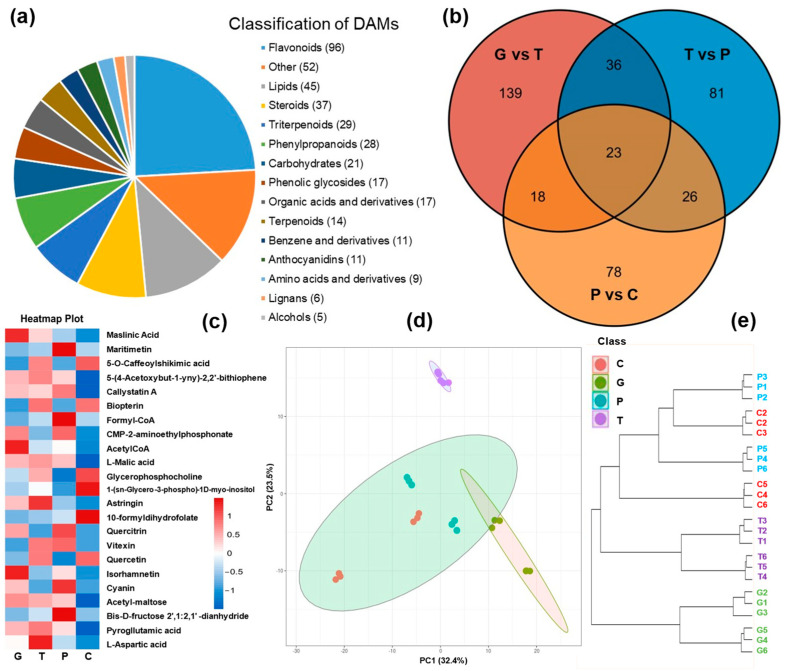
The metabolome analyses of *A. trifoliata* peels during ripening. (**a**) The classification of differentially accumulated metabolites (DAMs). (**b**) Venn diagram of DAMs. G: Green period; T: Transition period; P: Purple period; C: Cracking period. (**c**) Heat map of the common DAMs. The red segments indicate a relatively high expression level of DAMs, while the blue segments indicate a relatively low expression level of DAMs. (**d**,**e**) PCA score plot and dendrogram plot of DAMs. Metabolome analyses of all samples were conducted with six independent biological replicates.

In this study, a total of 398 differentially accumulated metabolites (DAMs) were identified among the compared samples (Table S1), categorized into 15 classes, with flavonoids, lipids, and steroids being the top three classes (Figure 2a). Among these metabolites, 23 DAMs were common to all three comparative analyses, mainly including quercetin, rutin, quercitrin, afzelin, citric acid, 2-furoic acid, phosphoric acid, malvidin-3-O-arabinoside, petunidin 3-galactoside, kaempferol-7-O-neohesperidoside, etc. (Figure 2b). The majority of these common DAMs belong to flavonoids, suggesting a strong association between flavonoid metabolism and the ripening and cracking of *A. trifoliata* (Figure 2c). Furthermore, PCA and hierarchical cluster analysis of the DAMs revealed clear distinctions among fruits at different developmental stages and demonstrated the repeatability of biological replicates (Figure 2d). The hierarchical cluster analysis not only supported the robust repeatability of the six biological replicates but also clearly grouped the purple and cracking periods into the same cluster (Figure 2e) [19]. After identification and analysis, these DAMs were further subjected to KEGG pathway analysis, and the 140 DAMs were matched to the KEGG databases. The biosynthesis of biosynthesis of plant hormones (map01060), and biosynthesis of terpenoids and steroids (map01062) were included in the top 20 KEGG pathways. The metabolome analysis showed that plant hormone metabolism might play an important role in the development and cracking of *A. trifoliata* fruit.

### 2.3. Overview of RNA-Seq of A. trifoliata Peel over the Ripening Stages

Predicting that the metabolite changes in *A. trifoliata* fruits during development and ripening are underpinned by alterations in gene expression, we conducted a comprehensive characterization of the fruit’s transcriptome. The number of unigenes in this study surpasses that obtained in previous research on this species (186,054 by Niu et al. [4], 11,749 by Yang et al. [9]). The expression levels of highly abundant detected genes (FPKM > 1000) in the twelve samples were analyzed (Figure S2). These highly abundant unigenes might play an important role in the development and cracking of *A. trifoliata* fruits, which mainly included dehydrin ERD14, ubiquitin C, major allergen, heat shock protein, pectinesterase inhibitor, 1-aminocyclopropane-1-carboxylate oxidase, ADP-ribosylation factor, xyloglucan endotransglucosylase/hydrolase (XTH), cytochrome p450, and salicylic acid-binding protein. Notably, the expression levels of heat shock protein, pectinesterase inhibitor, and XTH were significantly different among the different ripening periods, especially the pectinesterase inhibitor gene (*TR1887_c0_g4*) after cracking, which increased 27.95 times more than that of the green fruits. XTH is known to play a key role in the degradation of the cellulose xyloglucans/pectin network, contributing to fruit softening and cracking [11]. These results suggest a strong link between *A. trifoliata* fruit cracking and cell-wall metabolism. Additionally, 9795 unigenes were matched to the KEGG databases, encompassing 318 metabolic pathways, with 214 unigenes participating in plant hormone signal transduction. GO analysis revealed that most of the genes in the biological processes category were mainly involved in cellular processes, biological regulatory, and metabolic processes. And the cell, cell part, and organelle were the top-3 GO terms in the cellular components category. In terms of molecular functions, binding and catalytic activity were predominant (Figure S2).

### 2.4. Identification and Analysis of DEGs

In this study, differentially expressed genes (DEGs) in the three comparative groups (i.e., G vs. T, T vs. P, and P vs. C) were analyzed to explore the mechanism of A. trifoliata fruit cracking. A total of 8414 DEGs were identified in all three comparative groups (Table S2), with the G vs. T group having the largest number of DEGs (Figure S2). Notably, 380 DEGs were found in all three comparative analyses, including genes such as cytochrome P450, ferulate-5-hydroxylase, sugar/inositol transporter, pectate lyase, and cinnamyl alcohol dehydrogenase (Table S3), which were primarily associated with cell-wall and phenylpropanoid metabolisms [20].

Next, to gain insight into the dynamism of DEGs expression changes during ripening, a kinetic analysis was performed based on the FPKM data from G vs. T, T vs. P, and P vs. C. This analysis revealed four distinct kinetic clusters of co-expressed DEGs, characterized by significant transcriptional changes during the purple and cracking periods (Figure S2 and Table S4). Genes in cluster 3 and cluster 4 exhibited significant changes at the early stage, suggesting their association with the purple coloration of *A. trifoliata* fruit peel. Cluster 1 encompassed 1824 genes that initially displayed increased expression levels from the transition period to the purple period but then significantly decreased after cracking. Conversely, cluster 2 showed a gradual increase, with expression peaking in the cracking period (Figure S2 and Table S4) [21].

In this study, 200 TFs were found to be significantly differentially expressed during ripening, including 28 ERFs, 25 HLHs, and 10 WRKYs (Table S5), which may modulate the expression level of genes involved in fruit cracking. Among those differentially expressed TFs, ERF domain family members were the most abundant TFs, with most of the differentially expressed ERFs being down-regulated. The ERF transcription factor superfamily is an important regulator in plants, which mainly participate in various physiological processes, including plant growth and development, response to hormones, and abiotic stresses [22]. For example, ERFs are involved in the regulation of senescence-associated processes and the acquisition of mature fruit traits, such as those associated with ethylene signaling [10]. HLHs, another significant group of TFs, were differentially expressed during ripening. A total of 25 genes were encoded by the HLH family, of which 13 genes were up-regulated during ripening. This family of TFs is known to regulate carotenoid and anthocyanin biosynthesis in fruits like banana and sweet cherry [10,23]. Apart from the ERF and HLH genes, the Homeobox and WRKY families exhibited substantial differential expression during fruit ripening. The results were consistent with those reported by Yun et al. [11], who found ERF-, HLH-, and WRKY-related gene families to be the most abundant differentially expressed TFs when comparing transcriptome profiles of ripe and unripe pulp tissue in banana fruit. Although TFs play a crucial role in gene expression regulation, further investigation is needed to understand how these TFs regulate the cracking process in *A. trifoliata* fruit.

### 2.5. Joint Analysis of DAMs and DEGs

**Figure 3:**
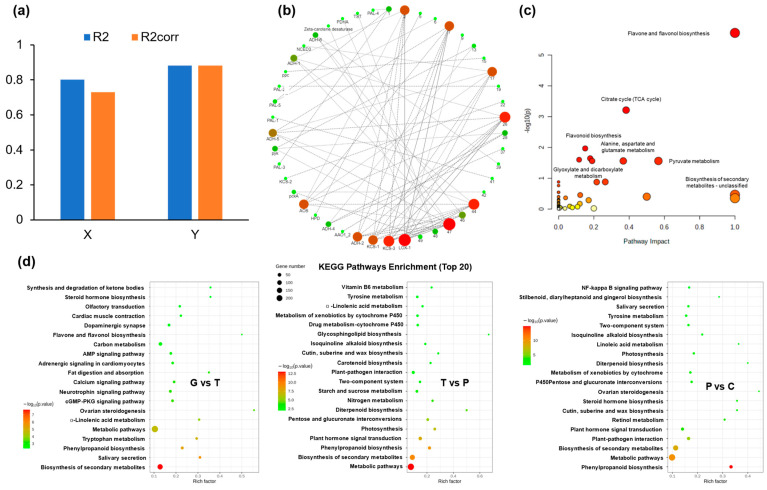
The integrated analyses of transcriptomics and metabolomics. (**a**) Overview plot of O2PLS integration performance. (**b**) The co-expression analysis of DEGs and DAMs based on Pearson correlation. (**c**–**d**) KEGG enrichment analysis of DAMs and DEGs. The vertical axis shows the top 20 enriched KEGG pathway terms. The size and color of the circle indicate the number of genes and *p* values of the enriched term, respectively. G: Green period, T: Transition period, P: Purple period, C: Cracking period.

The combination of RNA-seq and UHPLC-MS methods enabled the identification and annotation of numerous genes and metabolites involved in the ripening and cracking processes of *A. trifoliata* fruit. To gain a deeper understanding of the complex molecular processes and genetic regulation underlying these physiological changes, data integration was conducted using two-way orthogonal partial least squares (O2PLS), network correlation analysis, and KEGG enrichment analysis (Figure 3).

The intricate interplay among numerous metabolites and genes creates a complex network, posing challenges in our data analysis. Thus, an O2PLS model was used to decrease the noise and number of dominant correlations [24]. Figure 3a demonstrates the O2PLS model’s identification of two latent variables in the prediction dataset. These structures, predicting 88.1% of the total variation in transcription data and 73.1% in metabolite data, validate the model’s reliability. Following 1000 permutations for loading coefficient thresholds, 50 DEGs and 50 DAMs identified as most influential (Table S6), were subjected to Pearson’s correlation analysis. Notably, DAMs exhibited strong positive correlations with several DEGs, notably L-tryptophan, pyruvic acid, oxaloacetate, and glycogen. Subsequently, significant correlations (>0.8) between these elements were employed to construct network diagrams depicting their relationships in lipid and carbohydrate biosynthesis (Table S7, Figure 3b).

To evaluate the roles of DAMs and DEGs in the regulation of *A. trifoliata* fruit cracking, KEGG enrichment analysis was performed on the identified DAMs and DEGs. KEGG analysis of DAMs revealed that the flavone and flavonol biosynthesis, TCA cycle, and flavonoid biosynthesis were highly enriched (Figure 3c). The TCA cycle has been associated with phytohormone biosynthesis [6]. In addition, phenylpropanoid biosynthesis (ko00940) was shared in all three comparative groups (Figure 3d), indicating a strong connection between phenylpropanoid metabolism and the development and ripening of *A. trifoliata* fruit. KEGG analysis of DEGs showed that the photosynthesis and plant hormone signal transduction pathways were shared in both the T vs. P and P vs. C groups. Most DEGs were enriched in cell wall-related pathways, including pentose and glucuronate interconversions, amino sugar and nucleotide sugar metabolism, and galactose metabolism. Comparative analysis showed that the starch and sucrose metabolism, pentose and glucuronate interconversions were shared in both the T vs. P and P vs. C groups (Figure 3d).

### 2.6. Phenylpropanoid Metabolism

**Figure 4:**
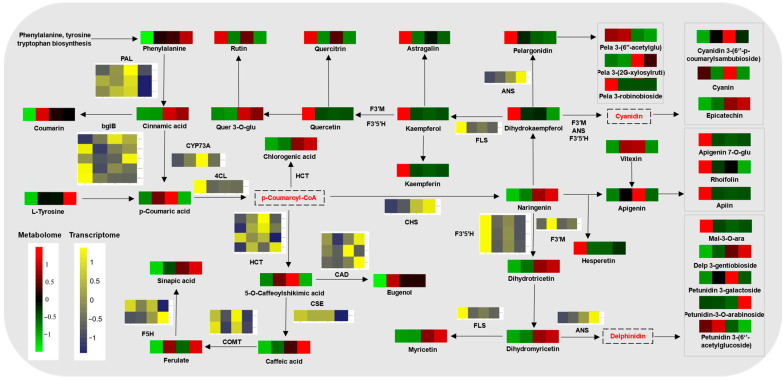
Regulatory network of phenylpropanoid biosynthesis of *A. trifoliata* peels during ripening. Differential metabolites are marked on the network in bright green (indicating down-regulation) and bright red (indicating up-regulation), and the heat maps above represent the G (green period), T (transition period), P (purple period), and C (cracking period) from left to right. The full name of each enzyme abbreviation is PAL, phenylalanine ammonia-lyase; ANS, anthocyanidin synthase; F5H, ferulate-5-hydroxylase; CHS, chalcone synthase; F3′5′H, flavonoid 3′,5′-hydroxylase; FLS, flavonol synthase; CYP73A, trans-cinnamate 4-monooxygenase; F3′M, flavonoid 3′-monooxygenase; HCT, shikimate O-hydroxycinnamoyl transferase; 4CL, 4-coumarate-CoA ligase; CAD, cinnamyl alcohol dehydrogenase. Metabolome and transcriptome analyses of all samples were conducted with six and three independent biological replicates, respectively.

Given the importance of phenylpropanoid metabolism, we reconstructed the network of phenylpropanoid pathways to predict the regulation of phenylpropanoid metabolism on the ripening and cracking of *A. trifoliata* fruits (Figure 4). Some DAMs, such as coumarin, cinnamic acid, and phenylalanine, are involved in the biosynthesis of phenylpropanoid, which serve as precursors for the synthesis of flavonoids [25]. Compared to the green period, these DAMs were generally higher in the other three ripening stages, except for p-coumaric acid, which decreased in the cracking period (Figure 4). Accumulation of flavonoids has been associated with improved plant tolerance to environmental stresses [26]. In the present study, it was observed that 98 flavonoid-related DAMs exhibited significant changes over different ripening periods, with quercetin and quercitrin being down-regulated. It has been reported that flavonoids play a role in scavenging reactive oxygen species (ROS) and detoxifying free radicals in plants, enhancing their ability to adapt to environmental changes [26]. In this study, FLS and F3′5′H, two corresponding genes, were also down-regulated. Therefore, we speculated that the down-regulation of flavonoids would result in the cracking of fruit by decreasing ROS scavenging.

To gain further insight into the accumulation mechanism of phenylpropanoids in *A. trifoliata* fruits, the expression levels of genes in the phenylpropanoid pathways were analyzed. As shown in Figure 4, the flavonoid 3′,5′-hydroxylase (F3′5′H) genes (*TR8303_c0_g1*, *TR18565_c0_g1*, and *TR33618_c0_g1*) were down-regulated throughout the ripening period. In contrast, the shikimate O-hydroxycinnamoyltransferase (HCT) genes (TR921_c0_g1, TR6655_c0_g1, and TR6165_c0_g1), anthocyanidin synthase (ANS) (TR1679_c0_g1), and trans-cinnamate 4-monooxygenase (CYP73A) (TR20874_c0_g1) genes, were inactive in the green period but were strongly activated in the transition and purple periods (Figure 4). These findings suggested that HCT, ANS, and CYP73A genes were the key genes involved in the purple pigmentation of *A. trifoliata* fruit peel. In addition, the F3′M (TR3989_c0_g1), ANS (TR1679_c0_g1), and CHS (TR5458_c0_g1) genes showed the highest expression after cracking. Based on these results, we assume that during the green period, high accumulations of early flavonoid precursors were induced by the strong activity of related structural genes (e.g., F3′5′H, PAL, and 4CL). However, during ripening, these structural genes in the early stages were silenced, and the accumulated metabolites were converted by ANS genes into the key anthocyanins involved in *A. trifoliata* fruit peel purpling (e.g., cyanidin 3-(6″-p-coumarylsambubioside), petunidin 3-galactoside, and delphinidin 3-gentiobioside).

Besides, some phenylpropanoids and related genes also participated in other important metabolic pathways associated with fruit cracking. For example, peroxidases can also participate in cell-wall rearrangement [27], while CAD and 4CL are involved in lignin biosynthesis [28], which is associated with changes in cuticle structure [12]. Notably, PAL, C4H, and 4CL are key genes in the early stages of lignin and flavonoid biosynthesis [29]. Ferulic acid and p-coumaric acid, along with CoA, are activated as feruloyl-CoA and p-coumaroyl-CoA, intermediate metabolites for the formation of monolignols and precursors for the acylation of xylan and lignin [30]. Lignin provides mechanical support and impermeability to the cell wall, and an increase in its content is related to an increase in ferulic acid and the induction of lignin biosynthetic genes during ripening. The regulation of phenylpropane metabolism can lead to changes in lignin and cuticle structure, thereby affecting cracking. In summary, phenylpropanoid metabolism plays a crucial role in the ripening and cracking of *A. trifoliata* fruit.

### 2.7. Photosynthesis Metabolism

Fruit ripening generally is accompanied by a reduction in photosynthesis transcript abundance and photosynthetic assimilate production [24]. In this study, genes related to photosynthesis were identified, and 18 of them exhibited down-regulation during ripening, with only two genes, ferredoxin-NADP^+^ reductase (petH) (*TR315245_c0_g1*) and the F-type H^+^-transporting ATPase subunit b (ATPF0B) (*TR315245_c0_g1*), showing the highest expression after cracking (Figure S3). Among these 20 DEGs, 7 genes were involved in PSI, 6 genes were in involved PSII, and 4 genes were linked to photosynthetic electron transport (Figure S3). Similarly, all eight DEGs involved in photosynthesis-antenna proteins were highly expressed in the green and transition periods but decreased in the purple and cracking periods. And all the genes were related to the light-harvesting chlorophyll protein complex (LHC) (Figure S3). Moreover, the transcriptome analysis was consistent with fruit-peel phenotypes during ripening. The genes involved in photosynthesis may be related to the peel color changes in *A. trifoliata*. The reduction in photosynthesis during fruit development led to a decrease in chlorophylls, and thus contributed to the peels’ color changing from green to purple. The results are in agreement with our previous results [31].

During ripening, DEGs involved in photosynthesis were down-regulated (Figure S3), suggesting a reduction in photosynthetic activity. Similarly, the transcript abundance of genes involved in starch and carbohydrate catabolism increased, and the levels of sucrose cleavage products decreased. This may be attributed to increased energy requirements in *A. trifoliata* fruit, typical of plant stress, or a mechanism by which fruits manipulate plant sugars to facilitate cracking. Moreover, it is well documented that the chemical composition and structure of the cuticle, as a key part of fruit peel, are closely related to fruit cracking [6,12]. The plant cuticle is a polymer composed of highly unsaturated fatty acids and lignin, of which linolenic acid is an unsaturated C18 fatty acid and the phenylpropanoids are the main precursors for lignin synthesis [12,20]. Previous studies reported that photosynthesis metabolism participated in cuticle formation in tomatoes [32] and litchi [12] by regulating fatty acid accumulation. The reduction in photosynthesis metabolism may result in decreased fatty acid content, impacting the chemical composition and structure of the cuticle, thereby reducing the mechanical strength of the *A. trifoliata* peel. Moreover, the top 20 pathways included three pathways associated with cuticle biosynthesis, indicating that the cuticle may play a significant role in *A. trifoliata* fruit ripening and cracking.

### 2.8. Plant Hormone Metabolism

A total of 54 DEGs were found to be involved in the plant hormone signal transduction pathway, encompassing all eight types of hormones (Figure S4). Notably, most differentially expressed signaling-related genes were linked with ABA and auxin-mediated signaling pathways. Auxin is known to modulate fruit development, while ABA is considered the main ripening regulator [22]. During ripening, the majority of the up-regulated genes were related to auxin signaling (Figure S4), suggesting that auxin may play a significant role in regulating *A. trifoliata* fruit’s ripening and cracking. Within the auxin signaling pathway, the two repressors of auxin-responsive transcription (i.e., AUX and IAAs) and the auxin response factor were the major molecular components [33]. In this study, six genes encoded IAA protein, with five genes displaying the highest expression in the green period. The decrease in IAA repressors induced the regulation of auxin-related genes, resulting in increased auxin levels during ripening, potentially contributing to fruit softening. In addition, the TIR1 gene (*TR5083_c0_g1*) increased during ripening, showing the highest expression level after cracking. It has been reported that auxin promotes fruit ripening [11,22], and our findings suggested that auxin signaling may be activated during ripening and cracking.

As shown in Figure S4, four ABA signaling-related genes were identified in *A. trifoliata* fruit peels, including two ABA receptors (PYL and PYR), serine/threonine-protein kinase SRK2 (SnRK2), and ABA-responsive element binding factor (ABF). The SnRK2 gene (*TR18749_c0_g1*) and the ABF gene (*TR1290_c2_g1*) showed increased expression levels after cracking. ABA is known to promote sugar accumulation in apples and citrus [22] and accelerate starch hydrolysis in tomatoes [26]. It appears that ABA signaling and auxin signaling are crucial for the ripening and cracking of the *A. trifoliata* fruit. However, detecting the eight types of plant hormones through untargeted metabolomics is challenging due to their low levels. Consequently, additional research is necessary to determine the concentrations of plant hormones in *A. trifoliata* fruit peels and to further investigate the impact of plant hormones on *A. trifoliata* fruit cracking.

### 2.9. Cell-Wall Metabolism

**Figure 5:**
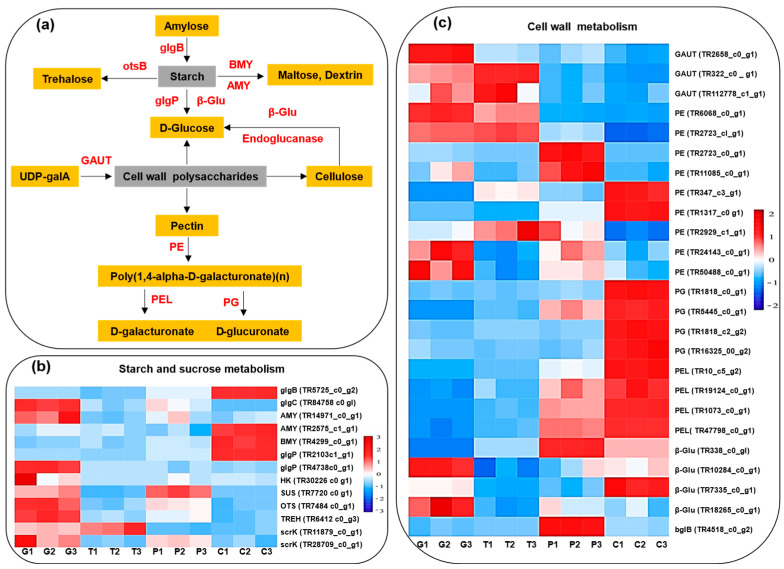
Simplified scheme of the polysaccharide metabolism pathway (**a**) and heat map diagram of the expression patterns for DEGs annotated for the starch and sucrose metabolism (**b**) and the cell wall analyzed metabolism (**c**) by KEGG. G: Green period, T: Transition period, P: Purple period, C: Cracking period. The red segments indicate a relatively high expression level of genes, while the blue segments indicate a relatively low expression level of genes.

Generally, fruit softening results from the depolymerization and solubilization of various cell-wall polysaccharides (e.g., pectin and hemicelluloses), alongside increased gene expression levels and enzyme activities related to cell-wall degradation [10]. In this study, a detailed analysis of cell wall-related genes provides insights into the cell-wall metabolism during ripening and cracking of *A. trifoliata* fruit. Two genes encoding endoglucanase were up-regulated during ripening (Figure 5), indicating an increase in endoglucanase expression, an enzyme associated with cellulose degradation and fruit softening [22]. Five genes encoding β-glucosidase displayed differential expression, with *TR338_c0_g1* and *TR7335_c0_g1* showing significantly higher FPKM values (Figure 5 and Table S2). Higher β-glucosidase levels in the purple and cracking periods may contribute to cellulose degradation and, consequently, fruit softening. All three genes related to GAUT, a key enzyme in pectin synthesis, were down-regulated during ripening, suggesting a decrease in pectin biosynthesis [10]. In contrast, all PL and PG genes exhibited the highest expression after cracking, indicating the activation of pectin-degrading enzymes, which is associated with cell-wall degradation and softening [22]. Nine genes encoding PE were differentially expressed, with *TR2723_c1_g1* and *TR24143_c0_g1* being activated at the early stage during fruit development, and *TR1317_c0_g1* and *TR347_c3_g1* being strongly activated after cracking. Higher expression of *TR1317_c0_g1* was particularly prominent in the cracking period (>500), suggesting that it plays a crucial role in pectin degradation. In conclusion, the transcriptome analysis demonstrates that the up-regulation of endoglucanase, β-glucosidase, PE, and PG genes after cracking is associated with the degradation of cellulose and pectin, which contributes to fruit softening and cracking.

Furthermore, the study demonstrates the enrichment of genes related to starch degradation during fruit cracking, including genes encoding glycogen phosphorylase (glgP), α-amylase (AMY), and β-amylase (BMY) (Figure 5 and Table S2). We observed a significant increase in expression levels of glgP genes and AMY genes during cracking. BMY (TR4299_c0_g1) expression also increased by 150% after cracking. These findings indicate significantly enhanced starch degradation during A. trifoliata fruit cracking.

### 2.10. Possible Explanations for A. trifoliata Fruit Cracking

Fruit cracking occurs when the stress on the fruit peel surpasses its strength [7]. *A. trifoliata* fruit, a kidney-shaped berry with a distinctive ventral suture, tends to crack along this suture upon reaching maturity in August [3]. Prior research by Niu et al. [4] emphasized the significant role of cell-wall metabolism in *A. trifoliata* fruit cracking, corroborating our study’s findings. In this study, the integrated transcriptomic and metabolomic analysis revealed that the cracking of *A. trifoliata* fruit was closely related to the phenylpropanoid, cuticle, plant hormone, and cell-wall metabolisms. From these findings, we developed a molecular mechanistic model to illustrate the ripening and cracking process of *A. trifoliata* fruit.

**Figure 6:**
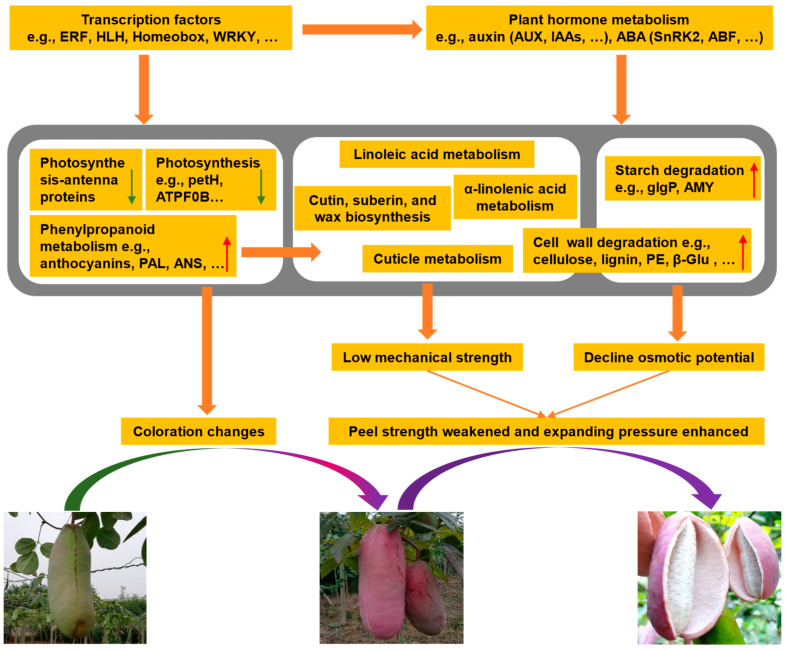
Scheme of the preliminary molecular mechanisms underlying the cracking of *A. trifoliata* fruits. The red and green arrows indicate the trend of changes in the color of *A. trifoliata* peel.

According to our model, the decrease in photosynthesis and the heightened phenylpropanoid metabolism, notably the accumulation of anthocyanins during late maturation, induce the peel’s color shift from green to purple (Figure 6). These changes also resulted in a decrease in fatty acid content and an increase in lignin content, which contributed to the regulation of the chemical composition and structure of the cuticle, ultimately reducing the mechanical strength of the *A. trifoliata* peel. Additionally, the observed up-regulation of genes related to cell-wall degradation and heightened expression levels of starch degradation genes collectively contribute to weakening the peel’s mechanical strength. The expression levels of hormone signal transduction-related genes, especially the ABA and auxin genes, were also found to be high, which may have directly accelerated peel growth or indirectly regulated other metabolisms, such as cell-wall degradation.

Overall, we propose that these DAMs and DEGs mentioned above, which are involved in the phenylpropanoid, photosynthesis, plant hormone, and cell-wall metabolisms, might change the chemical composition and structure of the cuticle, weaken the peel strength, and enhance the expanding pressure, ultimately leading to the *A. trifoliata* fruit cracking. This model enhances our understanding of the intricate mechanisms behind fruit ripening and cracking and offers insights that may prove beneficial in preventing fruit cracking in other crops.

## 3. Materials and Methods

### 3.1. Materials and Reagents

The *A. trifoliata* fruits utilized in this study were purchased from the Qianlin fruit and vegetable planting cooperative (Shanghai, China). Different developmental stages, namely green, transition, purple, and cracking periods (Figure 1a), were harvested every 10 days during ripening from 1 September to 1 October 2020. Fruits of uniform shape and size and without any signs of mechanical damage or fungal decay were selected for the study. All the chemicals and reagents were of analytical grade and purchased from Sinopharm Chemical Reagent (Shanghai, China).

### 3.2. Color and Chlorophyll Measurements

The color properties of *A. trifoliata* peels were determined using a LabScan XE color difference meter (HunterLab, Reston, VA, USA). The chlorophyll contents were measured according to the method of Jiang et al. [31]. Briefly, approximately 2 g of the peel sample was blended with 10 mL of 80% aqueous acetone (*v*/*v*) with continuous shaking at 4 °C for 30 min in the dark, then centrifuged at 6000× *g* for 10 min at 4 °C. The above process was repeated 2−3 times. The absorbances of supernatant were measured at 663 and 665 nm. Chlorophyll contents were calculated as follows:

$$Chlorophyll\;(ug/g)=\frac{\left(20.29\times A_{663}+8.05\times A_{645}\right)\times V}{M}$$

$$Chlorophyll\;a\;(ug/g)=\frac{\left(12.72\times A_{663}-2.59\times A_{645}\right)\times V}{M}$$

$$Chlorophyll\;b\;(ug/g)=\frac{\left(22.88\times A_{663}-4.67\times A_{645}\right)\times V}{M}$$

where *V* is the volume of the supernatant (mL), A is the absorbance, and M is the weight of the peel (g).

### 3.3. Peel Extract Preparations

Freeze-dried peel powder (~10 g) was placed in a separate container and mixed with 70% ethanol (1:10, *w*/*v*). The mixture was sonicated in an ultrasonic bath (S-10H, Zealway, Xiamen, China) at 4 °C for 1 h, followed by centrifugation at 8000× *g* for 10 min. A portion of the clear solution was used directly for metabolomics analysis, and the remainder was concentrated at 40 °C using a vacuum evaporator (RE-52AA rotary, Qingpu Huxi, Shanghai, China). The resulting extracts were freeze-dried and stored at −18 °C for the phytochemical constituent measurement.

### 3.4. Phytochemical Constituent Measurement

Total polyphenol content (TPC) was determined according to the methods of Jiang et al. [31] with slight modifications. Briefly, 0.2 mL of extract solutions at 0.25 mg/mL was mixed with 1.0 mL of 10% Folin–Ciocalteu phenol reagent and allowed to stand for 3 min and 0.8 mL of 75 mg/mL Na_2_CO_3_ was added, and the tube was shaken vigorously and incubated for 30 min in the dark at 37 °C. After cooling for 10 min at room temperature, the absorbance was measured at 765 nm using a microplate reader. TPC was expressed as gallic acid equivalents according to the equation obtained from a standard curve generated using pure gallic acid.

Total flavonoid content (TFC) was determined according to the methods of Jiang et al. [31] with slight modifications. Briefly, 0.5 mL of extract solutions at 0.25 mg/mL was mixed with 0.1 mL of 5% NaNO_2_. After 5 min, 0.1 mL of 10% Al (NO_3_)_3_ solution was added, and the mixture was allowed to stand for 6 min and then 1 mL 1 M NaOH was added. The reaction solution was mixed well and kept for 20 min at room temperature and the absorbance was determined at 510 nm. TFC was expressed as quercetin equivalents by comparison with a standard curve generated using pure quercetin.

Total anthocyanin content (TAC) of extracts was performed through the differential pH method as previously described by Jiang et al. [1]. Briefly, potassium chloride (0.2 M, pH 1.0) and sodium acetate (0.2 M, pH 4.5) were used as buffer solutions. The extracts were diluted with either pH 1.0 or pH 4.5 buffer solutions and absorbances were measured at 520 and 700 nm using a microplate reader. Anthocyanins in the extract were expressed as mg cyanidin-3-O-glucoside equivalents/100 g dry flower powder and calculated as follows:

Anthocyanins (mg/100 g) = A × MW × DF × 105/ε

where A is pH 1.0 (A520 nm–A700 nm) − pH 4.5 (A520 nm–A700 nm), MW is the molecular weight of cyanidin-3-O-glucoside (449.2 g/mol), DF is the dilution factor and ε is the molar extinction coefficient of cyanidin-3-O-glucoside (26,900 L/mol·cm^−1^).

### 3.5. Metabolomics Analysis and Data Processing

The UHPLC-QTOF-MS analysis was conducted by the Shanghai Jiao Tong University Analysis and Testing Center, following previously described procedures [31]. Raw data preprocessing involved peak alignment, extraction, normalization, deconvolution, and compound identification using Progenesis QI software (Version 2.2, Waters, Milford, MA, USA). To ensure stable variables, we applied a threshold of 30% for the relative standard deviation (RSD) of metabolites in the QC samples, establishing it as a criterion for assessing repeatability in the metabolomic datasets [17]. The retained peaks underwent normalization against the QC sample via quality control-based robust LOESS signal correction. MS/MS fragmentation spectra were utilized to determine fragmented molecule structures or compared with spectral data from available reference compounds in the HMDB (http://www.hmdb.ca) (accessed on 10 November 2021) and METLIN (http://metlin.scripps.edu/index.php) (accessed on 10 November 2021) databases. The significance of each metabolite to the OPLS-DA model was evaluated using the variable importance in projection (VIP) parameter. Metabolites meeting the criteria of VIP > 1, fold change ≥ 2 or fold change ≤ 0.5, and *p*-value ≤ 0.05 were identified as differentially accumulated metabolites (DAMs) for group discrimination.

### 3.6. Transcriptome Sequencing and Analysis

RNA samples were extracted from the four different periods of fruit using the three mean biological replicates studied above by metabolomics. RNA preparation, library construction, and sequencing were performed by Shanghai Applied Protein Technology Co., Ltd. (Shanghai, China), following previously described procedures [14]. The isolated RNA was assessed by 1% agarose gel electrophoresis and RNA bands were intact and intense (Figure S2), indicating good quality for library construction and sequencing. After removing low-quality reads, 760.88 million clean reads with Q30 percentages of 92.61–93.39% were selected for alignment to the reference genome sequence. This clean read assembly resulted in 255,884 unigenes, with lengths ranging from 201 bp to 16,677 bp. These sequence data have been deposited in the Sequence Read Archive (SRA) databases under accession number SUB13453700 [34]. Data generated from the Illumina/BGI platform were used for bioinformatics analysis. Genes with fold change ≥ 2 or ≤ 0.5 and p_adj_ < 0.05 were considered to be significantly differentially expressed genes (DEGs) using the DESeq2 R package (1.16.1) software. In addition, GO and KEGG pathway enrichment analysis of DEGs was performed with the R package cluster Profiler (https://bioconductor.org/*,* accessed on 1 December 2021).

### 3.7. Statistical Analysis

Data were analyzed by ANOVA and significant differences (*p* < 0.05) were determined by Duncan’s multiple range tests using SPSS 21.0 statistical data analytical software (Chicago, IL, USA). Integrated analysis of transcriptomic and metabolomic data included two-way orthogonal partial least squares (O2PLS), network correlation, and KEGG enrichment analysis (Figure S1), which were conducted using the OmicShare tool, a free online data analysis platform (https://www.omicshare.com/tools) (accessed on 1 March 2022).

## 4. Conclusions

The study investigated the mechanisms behind *A. trifoliata* fruit cracking by analyzing both the metabolome and transcriptome. A total of 398 DAMs and 8414 DEGs were identified during ripening and cracking. Notably, the most pronounced changes occurred in cell-wall metabolism, with 38 genes involved in this process. These genes encompassed five β-glucosidase genes, two endoglucanase genes, nine pectinesterase genes, two glgP genes, and two AMY genes. Most of these genes associated with cell-wall degradation and starch degradation displayed a significant up-regulation after cracking. Concurrently, the expression levels of 18 out of 20 genes related to photosynthesis metabolism showed a downward trend. These changes collectively contributed to alterations in the cuticle structure, ultimately leading to a reduction in cell-wall strength. Furthermore, this study identified 54 genes, particularly those involved in abscisic acid and auxin-related pathways, linked to phytohormone metabolism. Additionally, 200 transcription factors were identified, mainly from the ERF, HLH, and WRKY families, indicating their involvement in the regulation of cell-wall metabolism. These findings underscore the close relationship between *A. trifoliata* fruit cracking and cell-wall metabolism. Nevertheless, further research is required to delve into the specific mechanisms at play in this process.

## Data Availability

Data are contained within the article and Supplementary Materials.

## Supplementary Materials

The following supporting information can be downloaded at: https://www.mdpi.com/article/10.3390/ijms242316732/s1.
