# Supplementary material for: Integrative Metabolomic and Transcriptomic Landscape during Akebia trifoliata Fruit Ripening and Cracking
Source: Int J Mol Sci. 2023 Nov 24;24(23):16732. doi: 10.3390/ijms242316732 (PMC10706055; doi:10.3390/ijms242316732)
Supplement: Supplementary file 1 [file ijms-24-16732-s001.zip › Figures.pdf]

Figure legends

Figure S1. Overview of analytical approaches for integration of metabolomes and transcriptomes of *Akebia trifoliata* fruit. The integrative approach was applied to study *A. trifoliata* fruit metabolite and transcript fluctuations during ripening and cracking. Data processing methods allowed for the identification of transcripts and metabolites as single and integrated datasets to find biomarkers within each dataset and trends between them for a global understanding of the cracking in *A. trifoliata* fruit.

Figure S2. The transcriptome analyses of *A. trifoliata* peels during ripening.

(a) The expression profiles of abundant genes. The red segments indicate a relatively high expression level of genes, while the blue segments indicate a relatively low expression level of genes.

(b-c) GO and KEGG annotation classification of genes

(d) Venn diagram of differentially expressed genes (DEGs)

(e) Four clusters of co-expressed DEGs and their kinetic patterns.

Figure S3. The heat map illustrates the expression patterns of differentially expressed genes (DEGs) within the photosynthesis pathway (a) and the photosynthesis-antenna proteins (b) pathway in *A. trifoliata* peels during ripening.

G: Green period, T: Transition period, P: Purple period, C: Cracking period. The red segments indicate a relatively high expression level of genes, while the blue segments indicate a relatively low expression level of genes.

Figure S4. The heat map illustrates the expression patterns of differentially expressed genes (DEGs) within the plant hormone signal transduction pathway in *A. trifoliata* peels during ripening.

G: Green period, T: Transition period, P: Purple period, C: Cracking period. The red segments indicate a relatively high expression level of genes, while the blue segments indicate a relatively low expression level of genes.

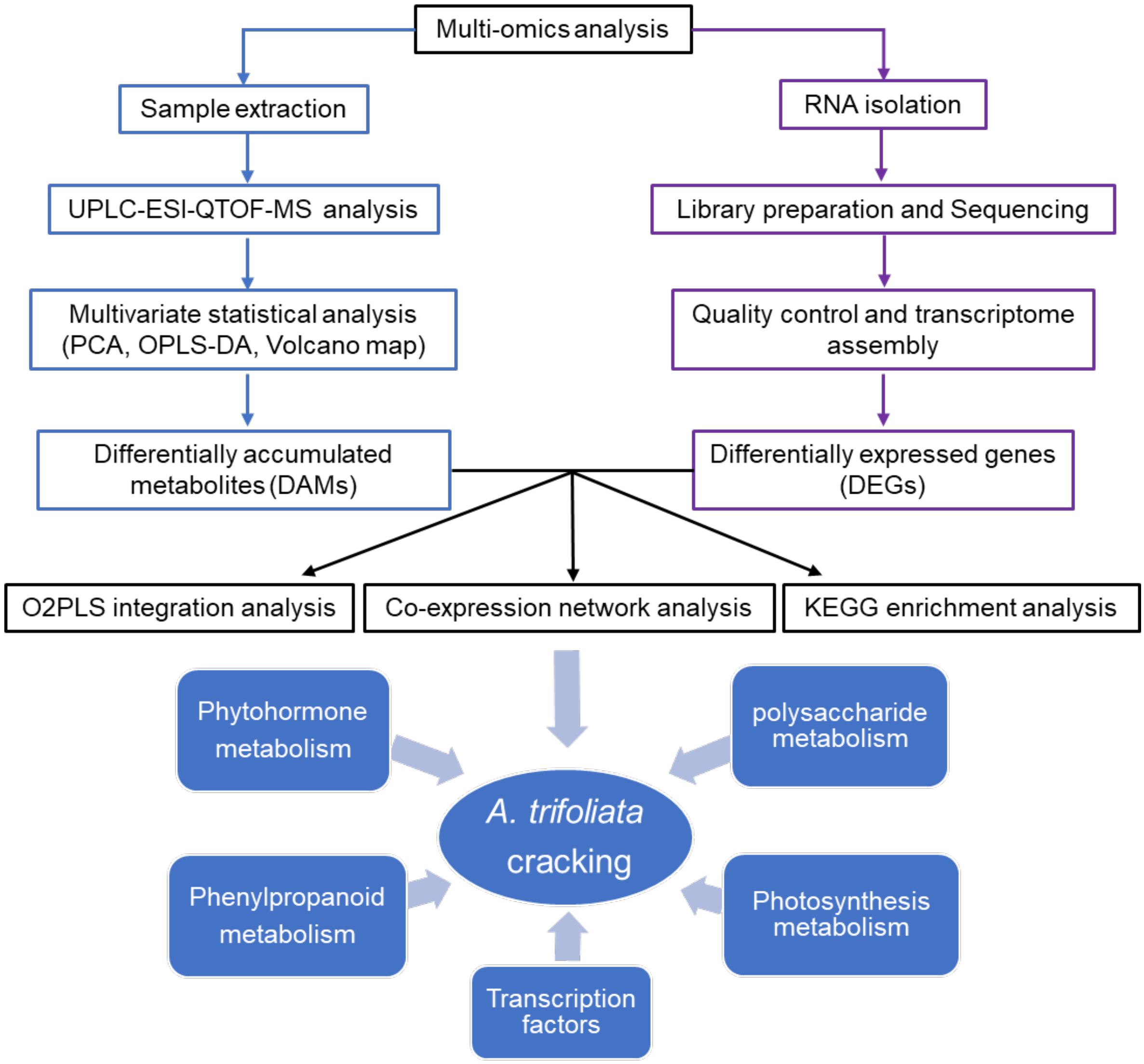
Multi-omics analysis
Sample extraction
RNA isolation
UPLC-ESI-QTOF-MS analysis
Library preparation and Sequencing
Multivariate statistical analysis (PCA, OPLS-DA, Volcano map)
Quality control and transcriptome assembly
Differentially accumulated metabolites (DAMs)
Differentially expressed genes (DEGs)
O2PLS integration analysis
Co-expression network analysis
KEGG enrichment analysis
Phytohormone metabolism
polysaccharide metabolism
A. trifoliata cracking
Phenylpropanoid metabolism
Photosynthesis metabolism
Transcription factors

Figure S1

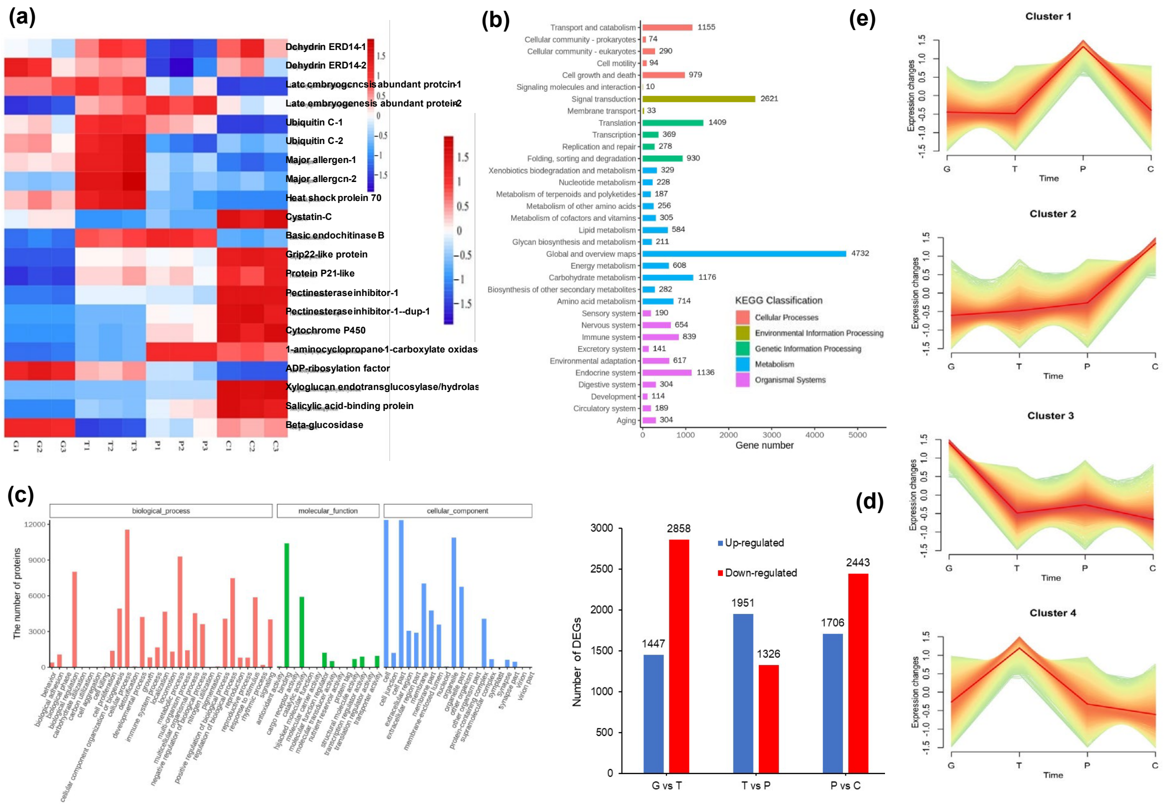
(a)
Dehydrin ERD14-1
Dehydrin ERD14-2
Late embryogenesis abundant protein-1
Late embryogenesis abundant protein-2
Ubiquitin C-1
Ubiquitin C-2
Major allergen-1
Major allergen-2
Heat shock protein 70
Cystatin-C
Basic endochitinase B
Grip22-like protein
Protein P21-like
Pectinesterase inhibitor-1
Pectinesterase inhibitor-1--dup-1
Cytochrome P450
1-aminocyclopropane-1-carboxylate oxidas
ADP-ribosylation factor
Xyloglucan endotransglucosylase/hydrolas
Salicylic acid-binding protein
Beta-glucosidase
G1 G2 G3 T1 T2 T3 P1 P2 P3 C1 C2 C3
1.5 1 0.5 0 -0.5 -1 -1.5
(b)
Transport and catabolism 1155
Cellular community - prokaryotes 74
Cellular community - eukaryotes 290
Cell motility 94
Cell growth and death 979
Signaling molecules and interaction 10
Signal transduction 2621
Membrane transport 33
Translation 1409
Transcription 369
Replication and repair 278
Folding, sorting and degradation 930
Xenobiotics biodegradation and metabolism 329
Nucleotide metabolism 228
Metabolism of terpenoids and polyketides 187
Metabolism of other amino acids 256
Metabolism of cofactors and vitamins 305
Lipid metabolism 584
Glycan biosynthesis and metabolism 211
Global and overview maps 4732
Energy metabolism 608
Carbohydrate metabolism 1176
Biosynthesis of other secondary metabolites 282
Amino acid metabolism 714
Sensory system 190
Nervous system 654
Immune system 839
Excretory system 141
Environmental adaptation 617
Endocrine system 1136
Digestive system 304
Development 114
Circulatory system 189
Aging 304
KEGG Classification
Cellular Processes
Environmental Information Processing
Genetic Information Processing
Metabolism
Organismal Systems
Gene number
(c)
biological_process
molecular_function
cellular_component
The number of proteins
(d)
Up-regulated
Down-regulated
Number of DEGs
1447 2858
1951 1326
1706 2443
G vs T
T vs P
P vs C
(e)
Cluster 1
Cluster 2
Cluster 3
Cluster 4
Expression changes
Time
G T P C

Figure S2

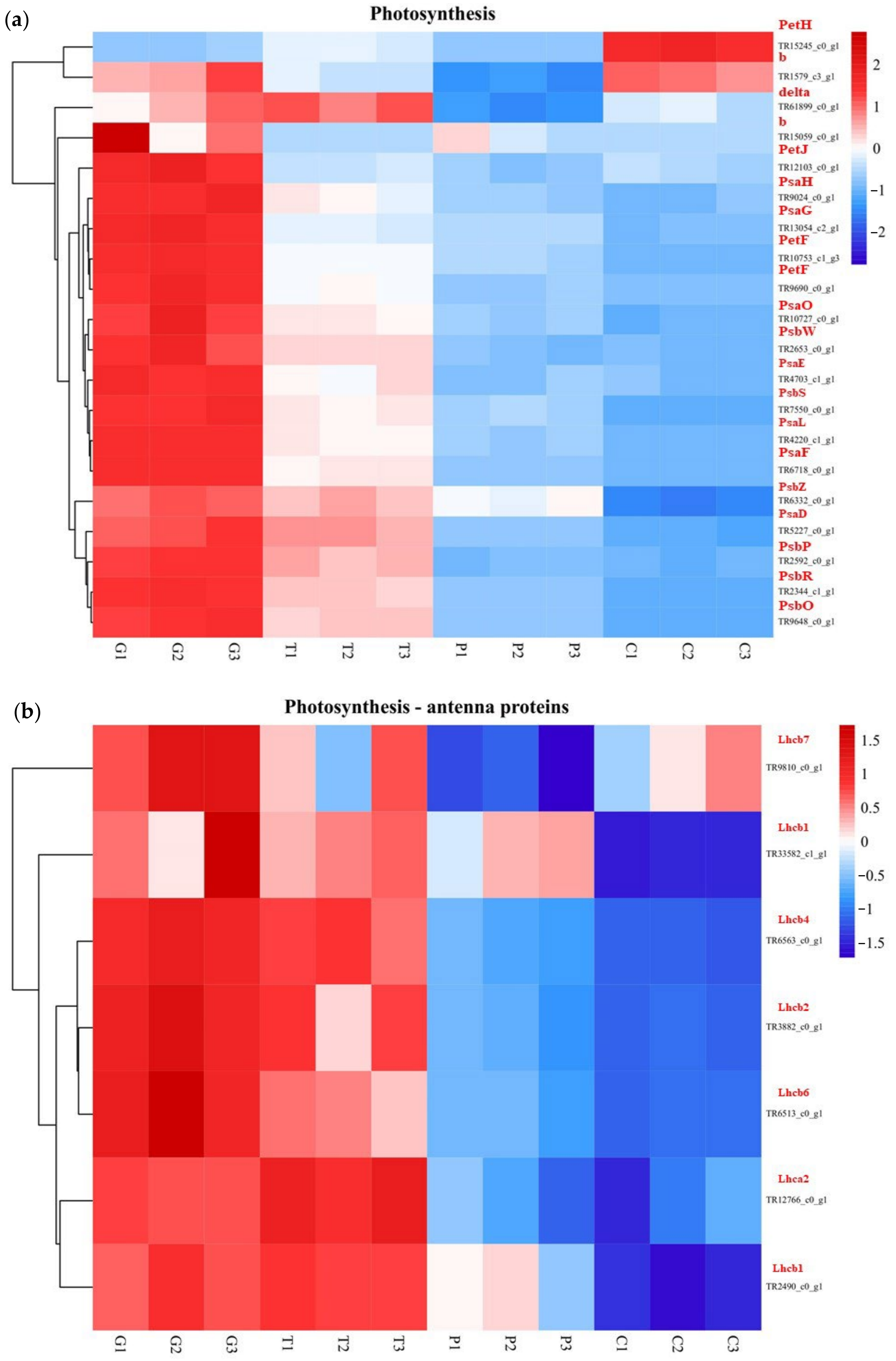

(a)
Photosynthesis
PetH
TR15245_c0_g1
b
TR1579_c3_g1
delta
TR61899_c0_g1
b
TR15059_c0_g1
PetJ
TR12103_c0_g1
PsaH
TR9024_c0_g1
PsaG
TR13054_c2_g1
PetF
TR10753_c1_g3
PetF
TR9690_c0_g1
PsaO
TR10727_c0_g1
PsbW
TR2653_c0_g1
PsaE
TR4703_c1_g1
PsbS
TR7550_c0_g1
PsaL
TR4220_c1_g1
PsaF
TR6718_c0_g1
PsbZ
TR6332_c0_g1
PsaD
TR5227_c0_g1
PsbP
TR2592_c0_g1
PsbR
TR2344_c1_g1
PsbO
TR9648_c0_g1
2
1
0
−1
−2
G1
G2
G3
T1
T2
T3
P1
P2
P3
C1
C2
C3
(b)
Photosynthesis - antenna proteins
Lhcb7
TR9810_c0_g1
Lhcb1
TR33582_c1_g1
Lhcb4
TR6563_c0_g1
Lhcb2
TR3882_c0_g1
Lhcb6
TR6513_c0_g1
Lhca2
TR12766_c0_g1
Lhcb1
TR2490_c0_g1
1.5
1
0.5
0
−0.5
−1
−1.5
G1
G2
G3
T1
T2
T3
P1
P2
P3
C1
C2
C3

Figure S3

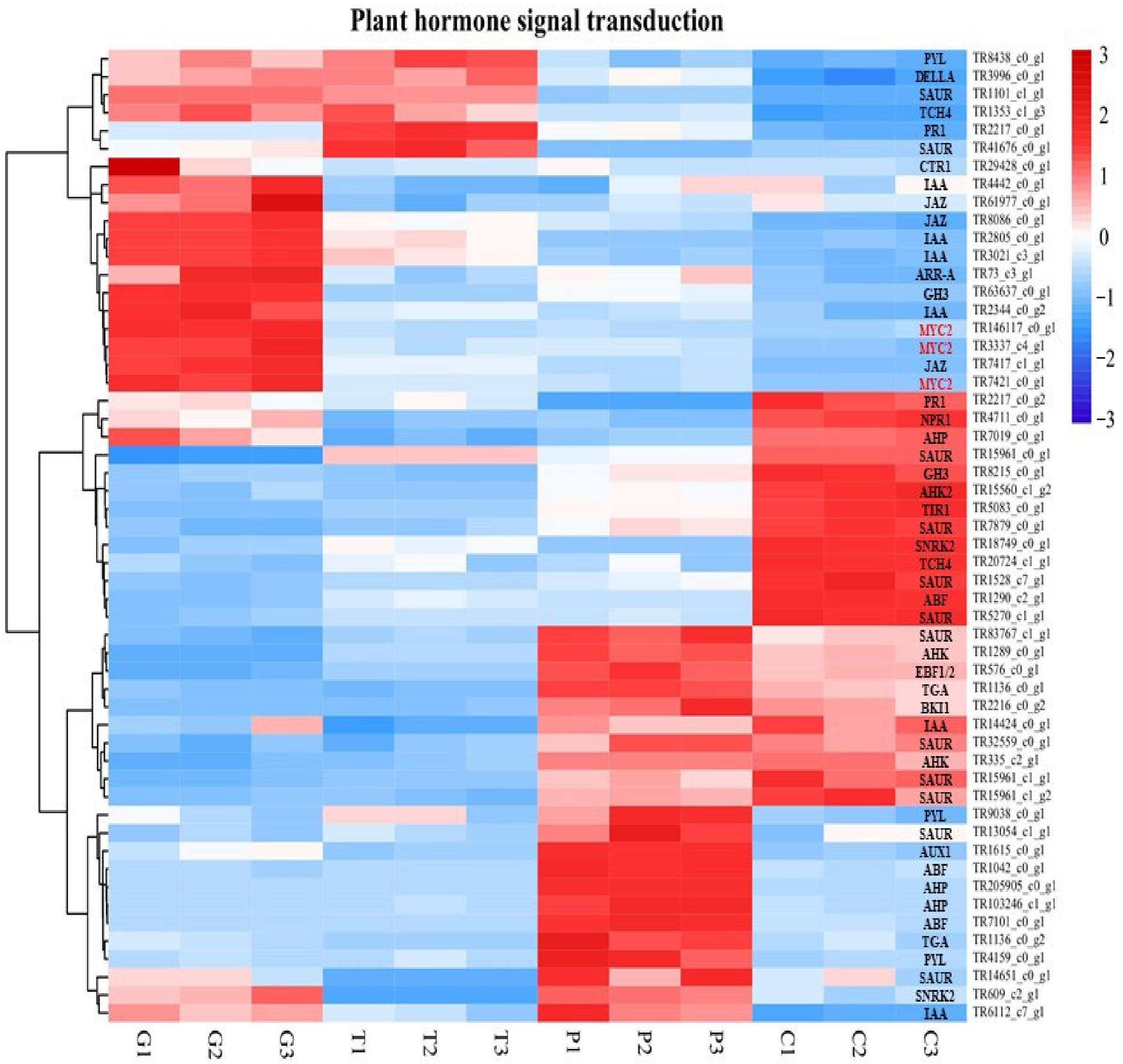

Plant hormone signal transduction
PYL TR8438_c0_g1
DELLA TR3996_c0_g1
SAUR TR1101_c1_g1
TCH4 TR1353_c1_g3
PR1 TR2217_c0_g1
SAUR TR41676_c0_g1
CTR1 TR29428_c0_g1
IAA TR4442_c0_g1
JAZ TR61977_c0_g1
JAZ TR8086_c0_g1
IAA TR2805_c0_g1
IAA TR3021_c3_g1
ARR-A TR73_c3_g1
GH3 TR63637_c0_g1
IAA TR2344_c0_g2
MYC2 TR146117_c0_g1
MYC2 TR3337_c4_g1
JAZ TR7417_c1_g1
MYC2 TR7421_c0_g1
PR1 TR2217_c0_g2
NPR1 TR4711_c0_g1
AHP TR7019_c0_g1
SAUR TR15961_c0_g1
GH3 TR8215_c0_g1
AHK2 TR15560_c1_g2
TIR1 TR5083_c0_g1
SAUR TR7879_c0_g1
SNRK2 TR18749_c0_g1
TCH4 TR20724_c1_g1
SAUR TR1528_c7_g1
ABF TR1290_c2_g1
SAUR TR5270_c1_g1
SAUR TR83767_c1_g1
AHK TR1289_c0_g1
EBF1/2 TR576_c0_g1
TGA TR1136_c0_g1
BKI1 TR2216_c0_g2
IAA TR14424_c0_g1
SAUR TR32559_c0_g1
AHK TR335_c2_g1
SAUR TR15961_c1_g1
SAUR TR15961_c1_g2
PYL TR9038_c0_g1
SAUR TR13054_c1_g1
AUX1 TR1615_c0_g1
ABF TR1042_c0_g1
AHP TR205905_c0_g1
AHP TR103246_c1_g1
ABF TR7101_c0_g1
TGA TR1136_c0_g2
PYL TR4159_c0_g1
SAUR TR14651_c0_g1
SNRK2 TR609_c2_g1
IAA TR6112_c7_g1
3
2
1
0
−1
−2
−3
G1 G2 G3 T1 T2 T3 P1 P2 P3 C1 C2 C3

Figure S4
